# How priming with body odors affects decision speeds in consumer behavior

**DOI:** 10.1038/s41598-023-27643-y

**Published:** 2023-01-12

**Authors:** Mariano Alcañiz, Irene Alice Chicchi Giglioli, Lucia A. Carrasco-Ribelles, Maria Eleonora Minissi, Cristina Gil López, Gün R. Semin

**Affiliations:** 1 Instituto de Universitario de Investigación Centrada en el Ser Humano (Human-Tech), Universitat Politécnica de Valencia, Valencia, Spain; 2 Fundació Institut Universitari per a la Recerca a l’Atenció Primària de Salut Jordi Gol i Gurina (IDIAPJGol), 08007 Barcelona, Spain; 3 Faculty of Social and Behavioral Sciences, Utrecht University, Utrecht, The Netherlands; 4 William James Center for Research, ISPA-Instituto Universitário, Lisbon, Portugal

**Keywords:** Human behaviour, Social behaviour

## Abstract

To date, odor research has primarily focused on the behavioral effects of common odors on consumer perception and choices. We report a study that examines, for the first time, the effects of human body odor cues on consumer purchase behaviors. The influence of human chemosignals produced in three conditions, namely happiness, fear, a relaxed condition (rest), and a control condition (no odor), were examined on willingness to pay (WTP) judgments across various products. We focused on the speed with which participants reached such decisions. The central finding revealed that participants exposed to human odors reached decisions significantly faster than the no odor control group. The main driving force is that human body odors activate the presence of others during decision-making. This, in turn, affects response speed. The broader implications of this finding for consumer behavior are discussed.

## Introduction

When purchasing a product, several factors play an important role. For instance, how much we are willing to pay (WTP) and the product or service value^1^. Of course, sensory information (e.g., visual, auditory, olfactory, gustatory, haptic, or a combination) contributes, although this type of information sometimes escapes conscious access. This is often the case with olfactory input^2^. Most of the research on odors has been performed with 'common odors' (e.g., floral scent, lavender, lemon). The study we report is, to our knowledge, the first examining the influence of human odors on possible purchasing behaviors. The human odors in question are chemosignals produced while experiencing an emotion (e.g., fear, happiness, and sweat produced during a resting state). Below, we give a brief overview of consumer behavior studies with common odors. Subsequently, we introduce research on human odors leading to the overview of the study reported here.

### Consumer behavior and common odors

Hirsch^3^ showed that olfactory priming affects WTP in an early study. He placed two identical Nike sneakers in two rooms in the experiment, one with a floral scent and the other with a neutral scent. Consumers in the scented floral room stated that they were 84% more likely to purchase the sneakers, with an average WTP of 10% more than consumers in the neutral room. In another study, Fiore, Yah, and Yoh^4^ exposed consumers to a sleepwear room containing a female mannequin, a threefold dressing mirror, two floral pillows, a white blanket, two white candles, and a vase with dried flowers. They had three scent conditions: unscented, "appropriate" scent, and "inappropriate scent." The WTP results revealed that consumers tended to spend more on the product with an appropriate scent (WTP = 29.6$) than with an inappropriate scent (WTP = 24.8$). Guéguen and Petr^5^ studied the difference between two scents (lavender and lemon) and an unscented condition in a restaurant for three Saturdays. The results showed that consumers in the lavender condition spent more (WTP = 21.1€) than consumers in the lemon (WTP = 18.1€) and unscented conditions (WTP = 17.5€).

The congruence and incongruence of gender-specific scents were examined by Spangenberg et al.^6^. They investigated the effect of dispersing the scents in both men's and women's clothing stores. Half of the consumers were exposed to a masculine scent (rose maroc) for two weeks, and the other half were exposed to a feminine scent (vanilla). Subjects in the congruent condition spent more than double the money as subjects in the incongruent condition (55.12$ vs. 23.01$). Other studies with more complex designs showed that congruency and incongruency of arousal levels influenced WTP^7–9^. For example, Michon et al.^8^ combined the arousal level induced by music (fast 96 bpm versus slow 60 bpm music tempo) and citrus scent vs. no scent in a corridor between two retail stores (one for food shopping and one for non-food shopping) in a shopping mall for four weeks. Consumers had spent more in the scent condition combined with the arousal induced by fast music than in the unscented condition combined with slow music arousal. Studies by Morrison et al.^9^ and Homburg et al.^7^ confirm that congruency can significantly influence WTP.

### Effects of human odors

The research we report here, namely the effects of human odors released while experiencing an emotion, can be seen potentially as falling in the field called biomarketing^10^. The sense of smell can be understood as a neurobiological system potentially activating certain body-related responses at different levels (i.e. neural and psychophysiological layers). For instance, perceived odors can automatically trigger psychophysiological reactions such as salivation, skin conductance, or changes in heart rate^11–13^. Moreover, odors can elicit other reactions escaping conscious access, such as arousing social/sexual attraction or stirring pleasant memories^14–16^. Given the social nature of on-site purchase activities, Bagozzi's principles of a neurobiological paradigm could lead to a novel conceptualization of the behavioral effects that body odors may have on consumer behavior.

We know that human body-related odors play an important role in social life^17,18^. Human chemosignals convey a great deal of biological and social information about people we interact with daily. Indeed, human odor provides reliable clues about gender^19^, age^20^, health/disease status^21^, the emotional states of others^22^, sexual orientation and even individual identity^23^. Indeed, one of the first things the sense of smell identifies about people nearby is their odor signature, which refers to odor signatures that convey someone's presence but are also unique markers of a person^24^.

Research with human chemosignals reveals that human body odors influence perception^25–28^. When human chemosignals (odors) are intense, then their conscious perception elicits an emotional reaction that is either one of likability or disgust (e.g., selection of a potential partner^29,30^ along with approach/withdrawal action tendencies^6^. In general, the perception of specific body odors escapes conscious access. For example, Zernecke et al.^27^ asked male participants to evaluate male facial expressions, from neutral to happy, under three different chemosignal conditions: exercise-related, anxious, and neutral sweats. The results showed that participants evaluated ambiguous happy faces as less happy in the anxiety sweat condition than in the other two conditions. Pause et al.^26^ reports similar results. In a recent study^25^ participants were primed with fear and neutral human body odors. They had to judge whether images of faces that were gradually becoming less blurred expressed emotion or not. The photos showed faces expressing fear, disgust, or anger or were neutral. The results suggest that participants identified fearful expressions as expressing an emotion faster than all the other expressions when exposed to fear odor (vs. neutral odor). Other studies have shown the influence of body odors in making spontaneous attributions of personality traits, with unpleasant aromas generally associated with socially undesirable traits^31–33^

### Human odors and consumer behavior: an experiment

Using human body-related scents to predict consumer behavior is entirely new. Current studies have only explored the behavioral effects of common odors on consumer perception and choices^34^. Body odors are potent triggers of sensory experiences and can regulate behavior in opposite directions, from approach to avoidance or happiness to disgust^30,35–37^.

A particular type of human chemosignal can modulate the quality of buyer–seller interactions. Although the literature on the influence of human body odors on behavior has grown lately^17,38–41^, research that investigates changes in consumers' buying decisions (e.g., WTP) evoked by human chemosignals have not been conducted yet. Unraveling this type of knowledge could induce a revolutionary change in the study of consumer purchase decisions and experience. The present study provides insights that help to clarify the impact of specific body-related odors on consumer WTP performance.

The current study was designed to examine the influence of human chemosignals collected in three emotion-inducing conditions: happiness, fear, and a relaxed condition (rest), aside from a control condition (no odor). We recorded participants' willingness to pay for a range of products and the speed with which they reached their decision. The general hypothesis was driven by the assumption that human odors, irrespective of the emotional condition under which they were produced, would not differentially affect the speed by which WTP decisions were reached. However, the overall speed of the decisions is only compared to the control condition (no human odor). This hypothesis was derived from earlier research^42,43^ that has shown that social chemosignals activate social and emotional areas of the brain relative to comparable common odors. We know from work done by Lundström et al.^42^ that "olfactory stimuli of high ecological relevance (i.e., human body odors) are processed by specialized neuronal networks similar to what has previously been demonstrated for auditory and visual stimuli". These studies show that human social odors, irrespective of the emotional context in which they were produced, activate socially dedicated brain circuits and implicitly signal the presence of others. We reasoned that irrespective of the conditions under which the human odor was produced (fear, happiness, rest), they would activate an implicit awareness of others' presence compared to the control condition in which no odor was presented.

How would activating the presence of others in the human odor condition—as opposed to the control condition (no odor)—affect the speed with which decisions to purchase? It is well known from the research on social facilitation that "dominant responses" (in other words, well-rehearsed or simple tasks)^44–46^ are facilitated by the presence of others. This means that in contexts that involve the performance of a well-rehearsed everyday activity, such as shopping, one would expect an enhanced performance when the presence of another is activated—even if implicitly. In other words, reaching decisions about what to pay should be faster in the presence of others compared to when nobody is present. In the case of complex tasks, where no dominant response has been established, performance would likely be impaired.

In our experiment, we used body odors, predicting that they would implicitly activate the presence of others. Therefore, we concluded and tested the prediction that human odors, irrespective of the emotional condition in which they are produced, should act as a facilitator when participants judge the price of a consumer object. They should judge prices much faster than participants in a control condition. We did not advance a differentiated hypothesis as a function of odor type, as we assumed that the relevant information was about implicitly registering the presence of another. As is well known, the processes induced by human odors escape conscious access^26,37,47,48^.

We examined the following hypotheses:

1. If the human body odors, irrespective of their production context (odor groups: fear, neutral, happiness), implicitly convey the presence of another person, then participants in the human odor conditions will reach decisions significantly faster than the control group.
2. If human chemosignals implicitly activate another's presence, then the production context of the chemosignal will not affect the speed with which people reach decisions.

## Results

**Figure 1:**
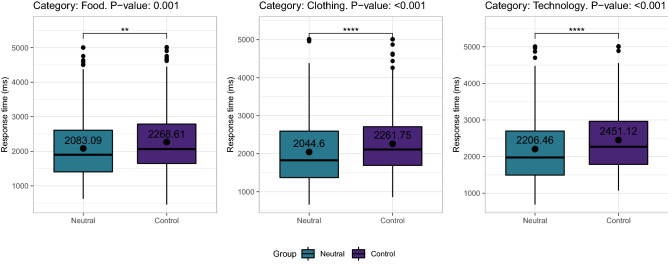
Response time differences between control participants and participants exposed to the neutral odor.

**Figure 2:**
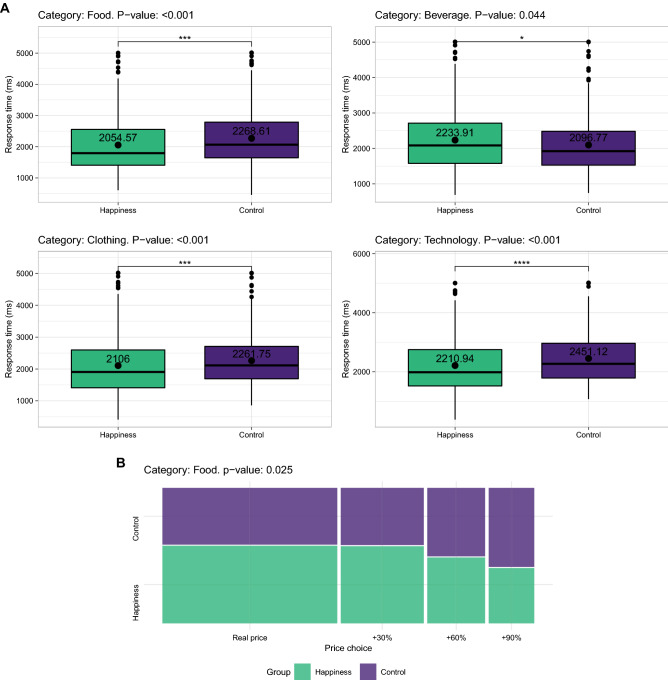
(**A**,**B**) Response time and price choice differences between control participants and participants exposed to the happiness odor.

**Figure 3:**
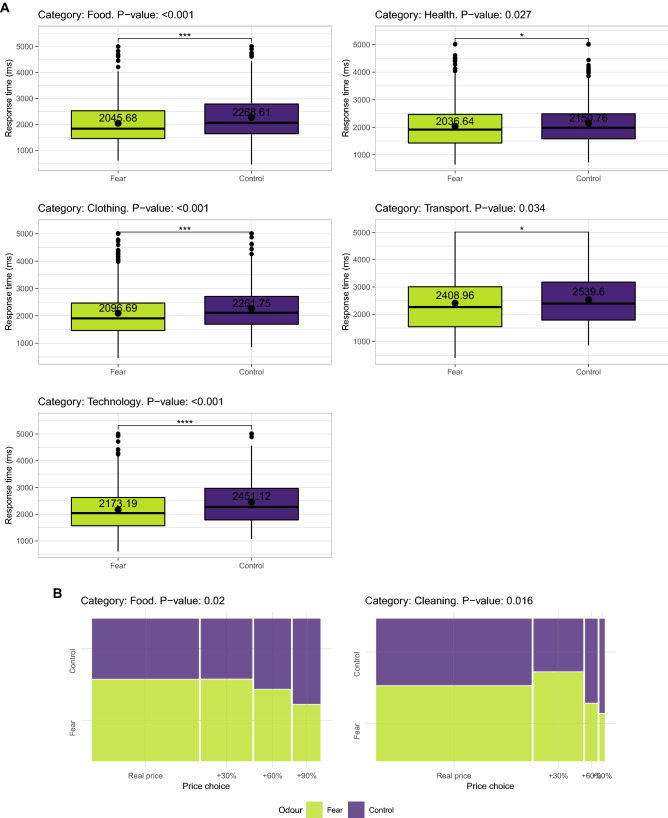
(**A**,**B**) Response time differences between control participants and participants exposed to the fear odor.

When comparing the responses of each odor condition individually to the control group (no odor), the following significant differences were found. When exposed to the neutral odor, participants chose a price more rapidly than control participants when the product belonged to the food, clothing, or technology categories (Fig. 1). When exposed to the happiness odor, participants chose the price more quickly when the product belonged to the food, clothing, beverage, or technology category (Fig. 2A). They also tended to choose closer to real prices than control participants in the food category (Fig. 2B). When exposed to the fear odor, participants chose the price more quickly than control participants when the product belonged to the food, clothing, health, transport, or technology category (Fig. 3A). They also tended to choose closer to real prices than control participants in the food and cleaning categories (Fig. 3B). The exact p values for the response time and price choice comparisons in all the categories can be found in tables S2 and S4 (see Appendix).

The control participants' responses were excluded to identify differences between odors in the second analysis. However, no significant differences were identified between the odors.

## Discussion

In the present study, we used body odors, predicting that they would implicitly activate the presence of others. This meant that human odors, irrespective of the emotional condition in which they are produced, should act as facilitators when participants judge a consumer object's price. Participants should judge prices much faster than participants in a control condition. We did not advance a differentiated hypothesis as a function of odor type, as we assumed that the relevant information was about implicitly registering the presence of another.

Overall, participants exposed to odors who chose a price more quickly tended to choose closer to real prices than control participants. Categories of products used daily, such as food, clothing, and technology, were the ones in which more differences were found between participants in the control and the experimental groups. We also considered whether the consumer categories we used would have a differential effect on judging speed. We did not advance a specific hypothesis here. However, we speculated that participants are likely to shop more frequently for food and clothing or spend more time examining and reading about technology products. Therefore, we surmised that such factors might play an important role in influencing which are dominant products and which are not. The three categories in the two groups seemed to produce faster responses in all three odor conditions. In addition, the food category produces the shortest response times, followed by clothing and then technology. Being everyday consumer goods, consumers likely know a priori their market value and the value that the participants themselves assume for each category, which is reflected in the speed of choice, regardless of the odor. However, the presence of odors, compared to the control group, seems to influence the speed of response, suggesting that the presence of a social variable acts as a filter for decision-making.

Furthermore, the results showed that several categories showed significant differences depending on the type of odor and the type of social variable. If in the case of the neutral odor, the three categories mentioned above showed faster choices, the happy odor encouraged faster choices in the beverage category with higher reaction times than in the control group, then the social variable of happiness is reflected in a choice with more weighted times. With the fear odor present, the categories health and transport were added to the initial three, with shorter choice times than the control group. Under the fear condition, the choice times of the health category showed faster choice times not only for the control group but also for the times of the other categories, suggesting that the social variable of fear seems to influence the speed of information processing in health products. It should be emphasized that the study was conducted after the first COVID-19 lockdown, in which the issue of hygiene took on absolute importance, and the fear of possible COVID-19 symptoms made people react more strongly to the purchase and consumption of hygiene products than before the pandemic. The transport category with the social variable of fear also influenced decision time, suggesting an association between the two variables.

On the other hand, our study found statistical differences for the variable "closeness to real price" in only two categories and two odors (food for happiness and food + cleaning for fear). Perhaps selecting a close-ended WTP survey instead of an open-ended one may explain this data. Potential improvements for future studies are detailed in the discussion section.

According to these results, we can confirm that if human body odors, irrespective of their production context (odor groups: fear, neutral, happiness), induce a state that implicitly conveys the presence of another person, then participants in the human odor conditions will reach decisions faster than the control group. Our results are in line with the previous one that revealed the influence of visual indicators of the presence of others on product valuation and WTP^49^.

In addition, even if the emotional states or their absence during the production of the chemosignals (odors) are not directly relevant to the task, there are still no differences in the speed with which prices are judged across the body odor conditions.

Several contributions have emerged from our research. First, the presented findings extend the research scope of scents as a reliable marketing tool at times that provides novel insights into the influence of different types of body-related odors on consumers' purchasing behaviors. Finally, the results confirm that the influence of body-related odors on customers' decision-making processes can be studied from a neurobiological approach^10^. Considering such a framework, the results of our study may be extrapolated to different selling contexts (e.g., services sector, tourism) wherein the presence of others is linked to unconscious mentalization of social behaviors—or what has come to be known as the theory of mind processes^10,50^—in terms of intentional states (e.g., needs, desires, beliefs, purposes, and feelings).

## Conclusions

Based on available research, it is evident that exogenous odor cues have a wide range of effects on consumers' decisions, eliciting both conscious and unconscious reactions to different scents. Body-related odors have not received as much attention in the context of consumer behavior despite neuroscientific research demonstrating the critical effect of body odor on social communication^18^. As the field matures, the social significance of human chemosignals is starting to gain more prominence in the study of consumer behavior, given the social nature of interactions between buyers and sellers^22^.

The primary goal of this research study was to explore the effects of body odors on consumers' WTP speed of performance. The reported results consistently confirm a relevant role of biological compounds found in specific body odors in consumer decision-making, which taps directly into an unconscious social representation concept: the presence of others. Specifically, we have shown that social information presented through human body odors implicitly activates the presence of others during decision-making, ultimately affecting response speed irrespective of the type of body odor. Notably, we demonstrated that the emotional state associated with body odors appears irrelevant at the 'managerial' level (i.e., speed of choices), which indicates that consumers appear to be less driven by sentiments when making decisions about WTP.

These findings shed new light on the role of human body-related scents in the context of consumer behavior, adding insightful evidence consistent with an emerging field of research within biomarketing. Finally, the study of the biological mechanisms of body odor perception, we believe, represents a further step in the field of consumer behavior. The use of neurobiological approaches may shed light on the rationality of hedonic choices, whether they involve a purchase decision, salesperson interaction decisions, or decisions made in situations of uncertainty. Therefore, future research should examine the facilitating effect of human body odors on different levels of consumer decision-making, such as those related to purchase intent and product recommendations to others. Further analyses could be conducted considering the possible confounding effect of socioeconomic factors such as gender and income level. Future improvements of this study could also include an open-ended direct WTP survey instead of a closed-ended one to analyze the influence of body odors on choosing lower or higher prices than the real ones.

## Methods

### Participants

Sixty-six participants were recruited, 26 of whom were controls. They were between 18 and 55 years old, righthanded, and nonsmokers. They were not using psychotropic drugs (including antidepressants, antipsychotics, anxiolytics, and mood stabilizers), were not pregnant or lactating, did not have any mental disorders, were not in psychological therapy, and were without severe psychotic symptoms (i.e., hallucinations or delusions).

Nine participants failed the Sniffin' Sticks screening test^52^ and were not considered for further analysis. The analysis included 31 subjects for the experimental condition group (age: 34.4 ± 9.4 years; 13 males, 18 females) and 26 subjects for the control condition group (age: 36.2 ± 8.6 years; 13 males, 13 females). A demographic description of the sample is available in Table S1 (see appendix).

Participants were randomly assigned to the three experimental conditions and a control condition based on a computer algorithm written in SAS^51^ and randomly allocated at a 1:1 ratio using randomly chosen block sizes. The experimental sample was balanced across the three within-subjects odor conditions.

Participants were recruited by a sample management company and received a financial reward for participation. Before the experimental session, they received information on the study procedure and gave written consent to be included in the study. The study received ethical approval from the Ethical Committee of the Polytechnic University of Valencia. Study procedures involving human participants were in line with the institutional and national research committee's ethical standards and the 1964 Helsinki Declaration and its later amendments.

### Procedure

Upon arrival at the laboratory, participants were led to a quiet experimental room and were given a seat on an adjustable chair. Participants were informed about the general experimental procedure and a written informed consent was obtained from all the participants. First, participants in the experimental group performed the two olfactory screening subtests of the Sniffin' Sticks test^52^ (for more details, see Appendix). Then, participants of both groups answered to a sociodemographic questionnaire, including information about gender, age, nationality, marital status, education, employment status, and income level.

First, regardless of the group participants belonged to, they performed nine practice trials of the price selection task. After that, participants performed a price selection task using a close-ended direct survey based on previous recommendations^53,54^. They were instructed to choose one of the four price options to indicate what they were likely to pay for the product. They were instructed to do so as accurately and as quickly as possible. In the practice trials, participants did not have any time restrictions. Participants proceeded with the experimental trials if there were no questions about the task requirements or instructions.

In the experimental trials, participants of both groups were presented with 108 products in a randomized order, each with four price options (standard price, a price increase of 30%, a price increase of 60%, and a price increase of 90%). They had to answer as quickly and accurately as possible within a five-second response window. Participants of the control group did the task once, while participants of the experimental group repeated the task for three times, each one with a different odor. The odor presentation was counterbalanced among participants, and product order was randomized in each task.

Participants in the experimental group were asked to place their head on a chin rest during practice and experimental trials. The dual purpose of the chin rest was to stabilize participants' heads and to ensure that their noses were at a constant distance (2 cm) from the odor vials, which was achieved by placing the odor vial into an adjustable clamp attached to the experimental table on the right side of the chin rest. Before the experimental phase, care was taken by the experimenters to ensure that participants of the experimental groups were in a comfortable position. In addition, participants of the experimental group took small breaks between odor conditions, having the chance to remove their head from the chin rest.

### Stimulus materials

Nine product categories were defined (Table 1). Each category contained 12 specific products, and each product was marked according to the market value price and three higher prices with three increments from the market value, namely, 30, 60, and 90%). Before the test trials, the experimenter placed the appropriate vial, a 60 mL polypropylene jar (Greiner Bio-One), without opening the lid. These were placed in the adjustable clamp. The olfactory stimuli (rest odor, fear odor, and happiness odor) were defrosted 30 min before odor exposure and were in the vial depending on the condition. The participant had a nose clip to prevent preliminary sniffing. Participants started the test trials once the vial lid was opened and the nose clip was removed. The experiment was conducted in a stand-alone manner. The odor procedure followed the previous works of Ref.^25^.

**Table 1:** Product categories.

Product category	Products
Food	Flour, salt, bread, rice, pasta, fruit, vegetables, water, meat, fish, coffee, pizza
Beverage	Beer, vodka, rum, pomace, gin, apple thief, fortified wine, vermouth, whiskey, red wine, white wine, rice liqueur
Cleaning products	Soap, toothpaste, shampoo, sponge, toilet paper, bleach, degreaser, household cleaner, anti-scale, glass cleaner, oval bucket, concentrated softener
Health products	Textile sanitizer, tame towel, disinfectant gel, mask, latex gloves, aloe cream, vitamins, sports watch, food supplements, lip balm, sunscreen, thermometer
Clothes	t-shirt/tank top, pants, shoes, socks, briefs/panties, scarf, polo, skirt, bikini/swimwear, suit, sneakers, sweatshirt
Domestic appliances	Washer, oven, dishwasher, vacuum cleaner, refrigerator, kettle, microwave, blender, food processor, tumble dryer, coffee maker, sandwich maker
Furniture	Table, chairs, sofa, lamp, bed, TV, glass table, mirror, wardrobe, shelf, doormat, footrest
Transport	Car, family car, sports car, bicycle, electric bicycle, scooter, motorcycle, scooter, skateboard, rollerblade, electric skateboard, electric wheel
Technology	Smartphone, pc, video games console, speaker, drone, headphones, action camera, Tablet, Alexa, projector, Smart TV, RV glasses

After the first experimental session was completed, the experimenter withdrew the vial and prepared the second vial. After a washout period of 5 min between vial sessions, the experimenter opened the lid of the second vial, and the above procedure was repeated for the subsequent two trial sessions.

### Statistical analysis

Two different analyses were performed after data processing (see Appendix). First, the response time and price selection were studied by individually comparing each experimental condition (i.e., one per odor) responses to the control group responses. Then, the response time and price selection from the experimental group in each condition were compared. These analyses were performed according to product category, aggregating the responses from the products that belonged to the same category.

To study the influence of the condition (i.e., experimental condition/control, or each odor) on the price choice, χ^2^ tests were performed. This test compares the expected and observed frequencies from each combination of price choice and condition. The results of these tests are shown together with a mosaic plot. Mosaic plots show the counts (observed frequencies) in contingency tables. The larger the area is, the more observations are clustered in the conditions that define it.

On the other hand, to study this influence on the response time, either Mann–Whitney tests or Kruskal–Wallis tests were performed depending on the number of conditions being compared. Kruskal–Wallis tests were followed by a post hoc Bonferroni adjusted Dunn analysis. The results of these tests are shown together with a boxplot, which also shows the mean value of the distribution. The normality of the data was previously checked using a Shapiro–Wilk test. The level of statistical significance was set as alpha < 0.05. The analysis was performed in R (version 4.0.4).

## Supplementary Information

Supplementary Information.

## Data Availability

The datasets generated during and/or analysed during the current study are available from the corresponding authors on reasonable request.
