# Supplementary material for: How priming with body odors affects decision speeds in consumer behavior
Source: Sci Rep. 2023 Jan 12;13:609. doi: 10.1038/s41598-023-27643-y (PMC9834684; doi:10.1038/s41598-023-27643-y)
Supplement: Supplementary file 1 — Supplementary Information. [file 41598_2023_27643_MOESM1_ESM.docx]

How Priming with Body Odors Affects Consumer Behavior

Mariano Alcañiz Raya^1^*, Irene Alice Chicchi Giglioli^1^, Lucia A. Carrasco-Ribelles^1,2^, Maria Eleonora Minissi^1^, Cristina Gil López^1^, Gün R. Semin^3,4^

# Appendix

## Screening test

The Sniffin’ Sticks test (Kobal et al., 1996) is an olfactory test composed of three subtests: the threshold test, the discrimination test, and the identification test. Subjects have to perform these tests without having drunk, eat and smoke in the last 15 minutes. The threshold test assesses the individual olfactory threshold. The discrimination test measures the olfactory ability to differentiate between smells, while the identification test aims to evaluate the ability to identify common smells. According to the present study's requirements, the threshold test and the discrimination test were administered to the experimental group. In the threshold test, participants were exposed to 16 triplets of Sniffin’ Sticks. Each triplet consisted of 2 blanks and one target stick presenting different dilutions of n-butanol. Triplets were presented every 30 seconds at 2 cm distance from participants’ nose, with 5 seconds of exposition to each Sniffin’ Stick, and 5 seconds of pause between sticks. The presentation order varied over triplets, following both increasing and decreasing orders with respect to the dilution amount of n-butanol in the target stick. Each time, triplets were presented two times in a different order to avoid casual responses. Participants’ olfactory threshold was computed following Kobal et al. (1996) instructions. In the discrimination test, 16 triplets were presented following the same procedure of the threshold test, except for the order and the double exposition to triplets. Each triplet consisted of two sticks with the same smell, and one target stick with a different smell. The discrimination score was the number of correct answers. Participants’ threshold and discrimination scores were compared with normative data of Oleszkiewicz et al., (2019), which settled the 10th percentile as the cut-off for normosomia.

## Data preprocessing

For each product assessed, the time taken to respond and the price chosen is available. First, we pre-processed the data to eliminate excessively short response times (<375ms). When the assessment exceeded 5 seconds, the next product was presented automatically. This could happen without the subject realising it, and they would select the price of the new product thinking that it was still the previous product. Thus, when an excessively short time (<375ms) was encountered, the price choice was moved from the immediately preceding product presented. The response time of that product was excluded from the analysis, along with the time and price choice of the current product. This happened in 0.86% of the assessments.

## Sample description

Demographic description of the sample is available below.

| **Variable** | **Control group  N = 26 (45.6%)** | **Experimental group N = 31 (54.4%)** | **P-value** |
| --- | --- | --- | --- |
| Age | 36.2 (8.60) | 34.2 (9.29) | 0.420 |
| Gender |  |  | 0.732 |
| Male | 13 (50.0) | 13 (41.9) |  |
| Female | 13 (50.0) | 18 (58.1) |  |
| Nationality |  |  | 0.006 |
| Spanish | 20 (76.9) | 31 (100) |  |
| Others | 6(23.1) | 0 (0.00) |  |
| Marital status |  |  | 0.512 |
| Non-independent single | 4 (15.4) | 9 (29.0) |  |
| Independent single | 7 (26.9) | 5 (16.1) |  |
| Married/Couple without children | 6 (23.1) | 9 (29.0) |  |
| Married/Couple with children | 7 (26.9) | 7 (22.6) |  |
| Divorced without children | 1 (3.85) | 0 (0.00) |  |
| Divorced with children | 1 (3.85) | 1 (3.23) |  |
| Education |  |  | 0.099 |
| Secondary school  (12 years) | 1 (3.85) | 0 (0.00) |  |
| Secondary school  (14 years) | 6 (23.1) | 9 (29.0) |  |
| Undergraduate  (18 years) | 6 (23.1) | 14 (45.2) |  |
| Graduate  (>18 years) | 13 (50.0) | 8 (25.8) |  |
| Employment |  |  | 0.220 |
| Employed | 16 (61.5) | 13 (41.9) |  |
| Self-employed | 3 (11.5) | 5 (16.1) |  |
| Student | 3 (11.5) | 7 (22.6) |  |
| Househusband/wife | 2 (7.69) | 0 (0.00) |  |
| Unemployed | 2 (7.69) | 6 (19.4) |  |
| Income level |  |  | 0.238 |
| <15.000€ | 8 (30.8) | 16 (51.6) |  |
| 15.001€-30.000€ | 13 (50.0) | 10 (32.3) |  |
| 30.001€-45.000€ | 3 (11.5) | 1 (3.23) |  |
| Not know/respond | 2 (7.69) | 4 (12.9) |  |

Table S1. Demographic description of the sample. Age is described as mean (standard deviation), while the rest of variables are described as N (%). The p-value corresponds to a t-test in the case of age, and a Chi-square test for the rest.

## Influence of each type of odour in response time relative to the absence of odours

|  |  |  |  |  | Analysis Reference group | | | | Analysis Condition group | | | |
| --- | --- | --- | --- | --- | --- | --- | --- | --- | --- | --- | --- | --- |
| **Category** | **Reference group** | **Condition group** | **p-value** | **Significance** | **Mean** | **SD** | **Median** | **IQR** | **Mean2** | **SD2** | **Median2** | **IQR2** |
| **Appliance** | Control | Happiness | 0.233 | ns | 2429.14 | 874.55 | 2293.00 | 1138.50 | 2391.49 | 992.15 | 2125.00 | 1369.00 |
| **Appliance** | Control | Fear | 0.263 | ns | 2429.14 | 874.55 | 2293.00 | 1138.50 | 2384.60 | 981.20 | 2236.50 | 1261.75 |
| **Appliance** | Control | Neutral | 0.575 | ns | 2429.14 | 874.55 | 2293.00 | 1138.50 | 2426.45 | 1015.10 | 2220.00 | 1367.00 |
| **Beverage** | Control | Happiness | 0.0438 | * | 2096.77 | 802.01 | 1921.00 | 955.25 | 2233.91 | 888.21 | 2085.00 | 1136.00 |
| **Beverage** | Control | Fear | 0.275 | ns | 2096.77 | 802.01 | 1921.00 | 955.25 | 2165.03 | 902.48 | 2103.50 | 1191.50 |
| **Beverage** | Control | Neutral | 0.229 | ns | 2096.77 | 802.01 | 1921.00 | 955.25 | 2163.67 | 845.94 | 2051.00 | 1109.00 |
| **Cleaning** | Control | Happiness | 0.559 | ns | 2007.49 | 744.88 | 1927.00 | 871.50 | 2022.18 | 907.37 | 1888.00 | 1282.00 |
| **Cleaning** | Control | Fear | 0.474 | ns | 2007.49 | 744.88 | 1927.00 | 871.50 | 2101.23 | 921.87 | 1947.00 | 1277.75 |
| **Cleaning** | Control | Neutral | 0.661 | ns | 2007.49 | 744.88 | 1927.00 | 871.50 | 2029.34 | 893.65 | 1878.00 | 1103.75 |
| **Clothing** | Control | Happiness | <0.001 | *** | 2261.75 | 804.34 | 2111.50 | 1017.25 | 2106.00 | 933.65 | 1905.00 | 1188.00 |
| **Clothing** | Control | Fear | <0.001 | *** | 2261.75 | 804.34 | 2111.50 | 1017.25 | 2096.69 | 906.70 | 1908.50 | 998.75 |
| **Clothing** | Control | Neutral | <0.0001 | **** | 2261.75 | 804.34 | 2111.50 | 1017.25 | 2044.60 | 920.66 | 1826.00 | 1222.00 |
| **Food** | Control | Happiness | <0.001 | *** | 2268.61 | 880.78 | 2064.50 | 1142.25 | 2054.57 | 915.37 | 1793.00 | 1147.00 |
| **Food** | Control | Fear | <0.001 | *** | 2268.61 | 880.78 | 2064.50 | 1142.25 | 2045.68 | 827.37 | 1839.00 | 1073.00 |
| **Food** | Control | Neutral | 0.00129 | ** | 2268.61 | 880.78 | 2064.50 | 1142.25 | 2083.09 | 920.29 | 1899.50 | 1207.25 |
| **Furniture** | Control | Happiness | 0.0584 | ns | 2318.15 | 860.22 | 2160.00 | 1041.00 | 2240.93 | 947.16 | 2008.00 | 1332.00 |
| **Furniture** | Control | Fear | 0.0698 | ns | 2318.15 | 860.22 | 2160.00 | 1041.00 | 2223.53 | 892.16 | 2022.00 | 1128.50 |
| **Furniture** | Control | Neutral | 0.145 | ns | 2318.15 | 860.22 | 2160.00 | 1041.00 | 2241.44 | 881.21 | 2039.00 | 1260.50 |
| **Health** | Control | Happiness | 0.498 | ns | 2150.76 | 776.43 | 1982.00 | 903.50 | 2144.35 | 897.65 | 1958.00 | 1229.00 |
| **Health** | Control | Fear | 0.0274 | * | 2150.76 | 776.43 | 1982.00 | 903.50 | 2036.64 | 826.51 | 1917.00 | 1038.00 |
| **Health** | Control | Neutral | 0.0699 | ns | 2150.76 | 776.43 | 1982.00 | 903.50 | 2093.58 | 908.00 | 1909.50 | 1174.50 |
| **Technology** | Control | Happiness | <0.0001 | **** | 2451.12 | 873.66 | 2270.00 | 1177.00 | 2210.94 | 880.27 | 1983.00 | 1229.25 |
| **Technology** | Control | Fear | <0.0001 | **** | 2451.12 | 873.66 | 2270.00 | 1177.00 | 2173.19 | 839.57 | 2040.50 | 1052.25 |
| **Technology** | Control | Neutral | <0.0001 | **** | 2451.12 | 873.66 | 2270.00 | 1177.00 | 2206.46 | 912.45 | 1977.00 | 1204.50 |
| **Transport** | Control | Happiness | 0.18 | ns | 2539.60 | 983.73 | 2394.50 | 1390.00 | 2446.22 | 999.33 | 2319.00 | 1374.50 |
| **Transport** | Control | Fear | 0.0337 | * | 2539.60 | 983.73 | 2394.50 | 1390.00 | 2408.96 | 1069.09 | 2265.00 | 1464.00 |
| **Transport** | Control | Neutral | 0.186 | ns | 2539.60 | 983.73 | 2394.50 | 1390.00 | 2451.28 | 1031.75 | 2279.00 | 1440.00 |

Table S2. p-values of the Mann-Whitney tests comparing response time between control participants and those in the different odour conditions. ns stands for non-significant.

## Comparisons of response time between odours

|  |  |  |  |  |  |  | Analysis Group 1 | | | | Analysis Group 2 | | | |
| --- | --- | --- | --- | --- | --- | --- | --- | --- | --- | --- | --- | --- | --- | --- |
| **Category** | **Test p-value** | **Test significance** | **Group 1** | **Group 2** | **Post-hoc p-value** | **Post-hoc significance** | **Mean** | **SD** | **Median** | **IQR** | **Mean2** | **SD2** | **Median2** | **IQR2** |
| **Appliance** | 0.842 | ns | Fear | Happiness | 0.97 | ns | 2384.60 | 981.20 | 2236.50 | 1261.75 | 2391.488 | 992.1487 | 2125 | 1369 |
| **Appliance** | 0.842 | ns | Fear | Neutral | 0.63 | ns | 2384.60 | 981.20 | 2236.50 | 1261.75 | 2426.451 | 1015.098 | 2220 | 1367 |
| **Appliance** | 0.842 | ns | Happiness | Neutral | 0.60 | ns | 2391.5 | 992.1 | 2125 | 1369 | 2426.451 | 1015.098 | 2220 | 1367 |
| **Beverage** | 0.591 | ns | Fear | Happiness | 0.35 | ns | 2165 | 902.5 | 2103.5 | 1191.5 | 2233.908 | 888.2117 | 2085 | 1136 |
| **Beverage** | 0.591 | ns | Fear | Neutral | 0.93 | ns | 2165 | 902.5 | 2103.5 | 1191.5 | 2163.675 | 845.9391 | 2051 | 1109 |
| **Beverage** | 0.591 | ns | Happiness | Neutral | 0.40 | ns | 2233.9 | 888.2 | 2085 | 1136 | 2163.675 | 845.9391 | 2051 | 1109 |
| **Cleaning** | 0.421 | ns | Fear | Happiness | 0.23 | ns | 2101.2 | 921.9 | 1947 | 1277.8 | 2022.178 | 907.3734 | 1888 | 1282 |
| **Cleaning** | 0.421 | ns | Fear | Neutral | 0.28 | ns | 2101.2 | 921.9 | 1947 | 1277.8 | 2029.335 | 893.6541 | 1878 | 1103.75 |
| **Cleaning** | 0.421 | ns | Happiness | Neutral | 0.90 | ns | 2022.2 | 907.4 | 1888 | 1282 | 2029.335 | 893.6541 | 1878 | 1103.75 |
| **Clothing** | 0.381 | ns | Fear | Happiness | 0.95 | ns | 2096.7 | 906.7 | 1908.5 | 998.75 | 2106.003 | 933.6451 | 1905 | 1188 |
| **Clothing** | 0.381 | ns | Fear | Neutral | 0.22 | ns | 2096.7 | 906.7 | 1908.5 | 998.75 | 2044.596 | 920.6598 | 1826 | 1222 |
| **Clothing** | 0.381 | ns | Happiness | Neutral | 0.24 | ns | 2106 | 933.6 | 1905 | 1188 | 2044.596 | 920.6598 | 1826 | 1222 |
| **Food** | 0.837 | ns | Fear | Happiness | 0.62 | ns | 2045.7 | 827.4 | 1839 | 1073 | 2054.572 | 915.3667 | 1793 | 1147 |
| **Food** | 0.837 | ns | Fear | Neutral | 0.97 | ns | 2045.7 | 827.4 | 1839 | 1073 | 2083.093 | 920.2879 | 1899.5 | 1207.25 |
| **Food** | 0.837 | ns | Happiness | Neutral | 0.59 | ns | 2054.6 | 915.4 | 1793 | 1147 | 2083.093 | 920.2879 | 1899.5 | 1207.25 |
| **Furniture** | 0.864 | ns | Fear | Happiness | 0.83 | ns | 2223.5 | 892.2 | 2022 | 1128.5 | 2240.932 | 947.1643 | 2008 | 1332 |
| **Furniture** | 0.864 | ns | Fear | Neutral | 0.74 | ns | 2223.5 | 892.2 | 2022 | 1128.5 | 2241.442 | 881.2131 | 2039 | 1260.5 |
| **Furniture** | 0.864 | ns | Happiness | Neutral | 0.59 | ns | 2240.9 | 947.2 | 2008 | 1332 | 2241.442 | 881.2131 | 2039 | 1260.5 |
| **Health** | 0.342 | ns | Fear | Happiness | 0.15 | ns | 2036.6 | 826.5 | 1917 | 1038 | 2144.35 | 897.6485 | 1958 | 1229 |
| **Health** | 0.342 | ns | Fear | Neutral | 0.65 | ns | 2036.6 | 826.5 | 1917 | 1038 | 2093.579 | 907.9966 | 1909.5 | 1174.5 |
| **Health** | 0.342 | ns | Happiness | Neutral | 0.33 | ns | 2144.3 | 897.6 | 1958 | 1229 | 2093.579 | 907.9966 | 1909.5 | 1174.5 |
| **Technology** | 0.954 | ns | Fear | Happiness | 0.80 | ns | 2173.2 | 839.6 | 2040.5 | 1052.3 | 2210.943 | 880.2686 | 1983 | 1229.25 |
| **Technology** | 0.954 | ns | Fear | Neutral | 0.99 | ns | 2173.2 | 839.6 | 2040.5 | 1052.3 | 2206.456 | 912.4541 | 1977 | 1204.5 |
| **Technology** | 0.954 | ns | Happiness | Neutral | 0.78 | ns | 2210.9 | 880.3 | 1983 | 1229.3 | 2206.456 | 912.4541 | 1977 | 1204.5 |
| **Transport** | 0.658 | ns | Fear | Happiness | 0.43 | ns | 2409 | 1069 | 2265 | 1464 | 2446.221 | 999.3306 | 2319 | 1374.5 |
| **Transport** | 0.658 | ns | Fear | Neutral | 0.43 | ns | 2409 | 1069 | 2265 | 1464 | 2451.276 | 1031.749 | 2279 | 1440 |
| **Transport** | 0.658 | ns | Happiness | Neutral | 1.00 | ns | 2446.2 | 999.3 | 2319 | 1374.5 | 2451.276 | 1031.749 | 2279 | 1440 |

Table S3. p-values of the Kruskal-Wallis tests followed by a post-hoc Bonferroni adjusted Tukey analysis comparing response time between the different odour conditions. ns stands for non-significant.

## Influence of each type of odour in price choice relative to the absence of odours and comparison between odours.

| **Product** | **Comparison** | **p-value** | **Significance** | **Real price Odor (%)** | **+30% Odor (%)** | **+60%   Odor (%)** | **+90%  Odor (%)** | **Real price Control (%)** | **+30% Control (%)** | **+60%   Control (%)** | **+90%  Control (%)** |
| --- | --- | --- | --- | --- | --- | --- | --- | --- | --- | --- | --- |
| **Appliance** | Happiness - Control | 0.831 | ns | 45.43% | 28.49% | 16.13% | 9.14% | 41.99% | 31.09% | 16.67% | 9.29% |
| **Appliance** | Fear - Control | 0.824 | ns | 42.47% | 31.72% | 15.86% | 7.26% | 41.99% | 31.09% | 16.67% | 9.29% |
| **Appliance** | Neutral - Control | 0.752 | ns | 44.35% | 27.15% | 16.94% | 9.95% | 41.99% | 31.09% | 16.67% | 9.29% |
| **Appliance** | Happiness- Fear - Neutral | 0.745 | ns |  |  |  |  |  |  |  |  |
| **Beverage** | Happiness - Control | 0.372 | ns | 46.24% | 30.91% | 14.25% | 7.80% | 48.08% | 26.60% | 13.14% | 10.90% |
| **Beverage** | Fear - Control | 0.116 | ns | 43.55% | 32.26% | 16.40% | 7.53% | 48.08% | 26.60% | 13.14% | 10.90% |
| **Beverage** | Neutral - Control | 0.345 | ns | 45.97% | 29.57% | 15.86% | 7.80% | 48.08% | 26.60% | 13.14% | 10.90% |
| **Beverage** | Happiness- Fear - Neutral | 0.965 | ns |  |  |  |  |  |  |  |  |
| **Cleaning** | Happiness - Control | 0.938 | ns | 71.77% | 18.28% | 6.72% | 2.42% | 71.47% | 17.95% | 7.05% | 3.21% |
| **Cleaning** | Fear - Control | 0.0163 | * | 67.74% | 25.54% | 4.03% | 1.34% | 71.47% | 17.95% | 7.05% | 3.21% |
| **Cleaning** | Neutral - Control | 0.341 | ns | 71.77% | 19.89% | 5.91% | 1.34% | 71.47% | 17.95% | 7.05% | 3.21% |
| **Cleaning** | Happiness- Fear - Neutral | 0.129 | ns |  |  |  |  |  |  |  |  |
| **Clothing** | Happiness - Control | 0.0579 | ns | 54.84% | 24.19% | 10.48% | 9.68% | 50.32% | 19.87% | 15.71% | 13.14% |
| **Clothing** | Fear - Control | 0.0837 | ns | 56.18% | 22.31% | 12.37% | 8.33% | 50.32% | 19.87% | 15.71% | 13.14% |
| **Clothing** | Neutral - Control | 0.00678 | ** | 57.80% | 22.85% | 10.75% | 6.99% | 50.32% | 19.87% | 15.71% | 13.14% |
| **Clothing** | Happiness- Fear - Neutral | 0.824 | ns |  |  |  |  |  |  |  |  |
| **Food** | Happiness - Control | 0.0255 | * | 50.81% | 23.92% | 14.25% | 9.41% | 44.23% | 21.15% | 17.63% | 16.03% |
| **Food** | Fear - Control | 0.0201 | * | 50.27% | 24.19% | 15.05% | 8.87% | 44.23% | 21.15% | 17.63% | 16.03% |
| **Food** | Neutral - Control | 0.0931 | ns | 47.04% | 23.39% | 15.32% | 9.68% | 44.23% | 21.15% | 17.63% | 16.03% |
| **Food** | Happiness- Fear - Neutral | 0.993 | ns |  |  |  |  |  |  |  |  |
| **Furniture** | Happiness - Control | 0.459 | ns | 51.34% | 26.61% | 11.29% | 9.14% | 49.04% | 25.00% | 13.14% | 12.50% |
| **Furniture** | Fear - Control | 0.642 | ns | 51.34% | 26.88% | 10.75% | 10.75% | 49.04% | 25.00% | 13.14% | 12.50% |
| **Furniture** | Neutral - Control | 0.295 | ns | 48.66% | 29.84% | 11.29% | 9.14% | 49.04% | 25.00% | 13.14% | 12.50% |
| **Furniture** | Happiness- Fear - Neutral | 0.928 | ns |  |  |  |  |  |  |  |  |
| **Health** | Happiness - Control | 0.202 | ns | 70.97% | 17.20% | 8.06% | 2.42% | 72.12% | 16.03% | 5.45% | 4.81% |
| **Health** | Fear - Control | 0.256 | ns | 70.97% | 18.55% | 6.99% | 2.42% | 72.12% | 16.03% | 5.45% | 4.81% |
| **Health** | Neutral - Control | 0.0812 | ns | 71.24% | 18.55% | 6.72% | 1.61% | 72.12% | 16.03% | 5.45% | 4.81% |
| **Health** | Happiness- Fear - Neutral | 0.959 | ns |  |  |  |  |  |  |  |  |
| **Technology** | Happiness - Control | 0.0688 | ns | 54.30% | 25.00% | 9.68% | 9.68% | 50.32% | 22.44% | 16.35% | 8.65% |
| **Technology** | Fear - Control | 0.713 | ns | 50.00% | 22.58% | 13.71% | 10.48% | 50.32% | 22.44% | 16.35% | 8.65% |
| **Technology** | Neutral - Control | 0.341 | ns | 55.38% | 23.12% | 11.83% | 9.14% | 50.32% | 22.44% | 16.35% | 8.65% |
| **Technology** | Happiness- Fear - Neutral | 0.616 | ns |  |  |  |  |  |  |  |  |
| **Transport** | Happiness - Control | 0.153 | ns | 51.08% | 24.19% | 13.44% | 10.48% | 55.77% | 24.04% | 12.82% | 5.77% |
| **Transport** | Fear - Control | 0.0613 | ns | 48.92% | 22.58% | 17.47% | 9.68% | 55.77% | 24.04% | 12.82% | 5.77% |
| **Transport** | Neutral - Control | 0.559 | ns | 50.00% | 25.27% | 12.63% | 7.80% | 55.77% | 24.04% | 12.82% | 5.77% |
| **Transport** | Happiness- Fear - Neutral | 0.488 | ns |  |  |  |  |  |  |  |  |

Table S4. p-values of the χ2 tests performed to study the differences in price choice between conditions (i.e., control VS one experimental condition, or between the three experimental conditions). ns stands for non-significant.
